# Interleukin-6 as an enhancer of anti-angiogenic therapy for ovarian clear cell carcinoma

**DOI:** 10.1038/s41598-021-86913-9

**Published:** 2021-04-08

**Authors:** Toshiyuki Seki, Nozomu Yanaihara, Jason Solomon Shapiro, Misato Saito, Junya Tabata, Ryo Yokomizo, Daito Noguchi, Takafumi Kuroda, Ayako Kawabata, Jiro Suzuki, Kazuaki Takahashi, Haruka Matsuzawa, Misayo Miyake, Masataka Takenaka, Yasushi Iida, Satoshi Yanagida, Aikou Okamoto

**Affiliations:** 1 Department of Obstetrics and Gynecology, The Jikei University School of Medicine, 3-25-8 Nishi-Shinbashi, Minato-ku, Tokyo, 105-8461 Japan; 2 Feinberg Cardiovascular Research Institute, Northwestern University, Chicago, IL 60611 USA; 3 Department of Pathology, The Jikei University School of Medicine, Tokyo, Japan

**Keywords:** Ovarian cancer, Cancer microenvironment

## Abstract

Ovarian clear cell carcinoma (OCCC) is a subtype of epithelial ovarian cancer (EOC) that is associated with elevated interleukin-6 (IL-6) expression, resistance to chemotherapy, and increased mortality. Although bevacizumab (Bev) is a widely used anti-angiogenic agent for EOC, the efficacy of Bev and the role of IL-6 in modulating angiogenesis in OCCC are unknown. We performed tube formation assays using human umbilical vein endothelial cells (HUVEC) cultured in OCCC cell-conditioned medium and using cells directly co-cultured with OCCC cells. We observed that IL-6 inhibition significantly mitigated the ability of Bev to impede tube formation in both cases. Furthermore, IL-6 blockade disrupted the anti-angiogenic efficacy of Bev and its concomitant anti-tumor activity. In addition, IL-6 inhibition resulted in a significant increase in angiopoietin-1 (Ang1) secretion and decreased vascular endothelial growth factor (VEGF) expression. Clinical specimens also exhibited this reciprocal relationship between IL-6 and Ang1 expression. Finally, depletion of Ang1 abrogated the effects of IL-6 inhibition on Bev activity, demonstrating that IL-6 supports the anti-angiogenic activity of Bev by suppressing Ang1 expression and promoting dependence on VEGF for angiogenesis. Altogether, our data suggest that OCCC tumors with high IL-6 levels are candidates for Bev therapy.

## Introduction

Among the five distinct histotypes of epithelial ovarian cancer (EOC), ovarian clear cell carcinoma (OCCC) exhibits unique biological and molecular features, and is therefore recognized as a distinct entity presenting unique challenges for treatment^1^. The prevalence of OCCC varies geographically; while accounting for only 1–12% of EOC cases in Western countries, OCCC is relatively frequent among Asian EOC patients^2^. Until recently, few clinical trials have examined OCCC specifically and there is insufficient evidence regarding optimal chemotherapeutic approach, including molecular medicine. In general, OCCC is less sensitive to platinum-based first-line chemotherapy than other EOC histotypes and is associated with poor prognosis in advanced cases^3^. Identification of novel molecular targets related to carcinogenesis may help in patient stratification, prognosis, and treatment decisions. The AT-rich interactive domain 1A gene (*ARID1A*) and the phosphatidylinositol 4,5-bisphosphate 3-kinase catalytic subunit α gene (*PIK3CA*) are frequently mutated in OCCC, and co-mutation in transgenic mice promotes OCCC tumor initiation, possibly by continuous stimulation of interleukin-6 (IL-6)-dependent pro-inflammatory and pro-tumorigenic signaling as IL-6 is elevated in OCCC patients^4^. Moreover, the potential involvement of elevated IL-6/signal transducer and activator of transcription 3 (STAT3) pathway activity in OCCC pathogenesis has been reported by several groups^5,6^ and so may be a promising therapeutic target.

The anti-angiogenic drug bevacizumab (Bev), a monoclonal antibody against human vascular endothelial growth factor (VEGF), has been incorporated into first-line chemotherapy and follow-up maintenance therapy for advanced EOC. Although a randomized phase III trial evaluating the clinical benefits of Bev for EOC treatment (GOG-0218) did not show improvement in overall survival^7^, a recent retrospective plasma sample biomarker analysis from GOG-0218 patients found that high serum IL-6 concentration was predicted to have better anti-angiogenic treatment efficacy^8^. Thus, serum IL-6 may help to identify patients most responsive to anti-angiogenic treatment. Further, these findings suggest that IL-6 signaling may potentiate the anti-angiogenic actions of VEGF blockade by Bev.

In this study, we examined the modulatory actions of IL-6 on Bev anti-angiogenic efficacy by conducting tube formation assays in co-cultures of OCCC cell lines and human umbilical vein endothelial cells (HUVEC) in the presence or absence of Bev and/or anti-IL-6 antibody. Results suggested that IL-6 promotes the anti-angiogenic efficacy of Bev by suppressing angiopoietin-1 (Ang1) release. Further, we present evidence that IL-6 also suppresses Ang1 production in human OCCC tissue. Collectively, these results provide valuable information for the development of individualized OCCC treatment strategies.

## Results

### Attenuation of Bev anti-angiogenic efficacy by IL-6 signal blockade

**Figure 1:**
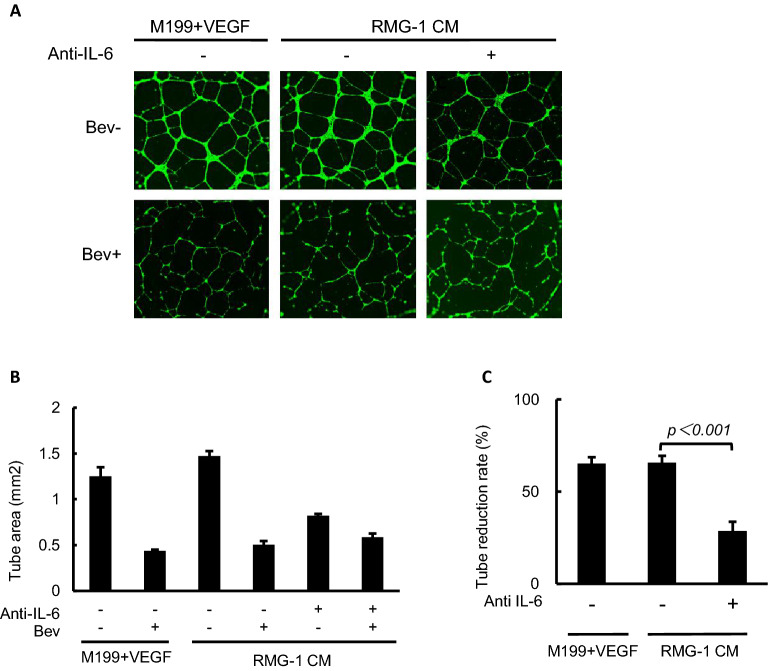
Attenuation of the anti-angiogenic function of Bev in culture media of IL-6 blocked OCCC cell. (**A**) Representative images of tube formation of HUVEC with the indicated culture media and reagents (magnification × 100). Fluorescent microscope observation was made after 18 h of incubation and Calcein AM staining. (**B**) Tube areas in each well with indicated condition were measured by hybrid cell count software. Data were average of triplicated well. (**C**) The tube reduction rate by Bev treatment with or without IL-6 blockade. IL-6 blockade weakened Bev function. Data are shown from one of two independent experiments with similar results. Error bars are SEs. All the images were observed by fluorescence microscope (BZ-X800, Keyence, Osaka, JAPAN). Then, these obtained images were analyzed by the BZ-H4C analytic application (Keyence) for hybrid cell count and the BZ-H4CM application (Keyence) for macro cell count.

To investigate the influence of IL-6 signaling on anti-angiogenic therapy in OCCC, we first assayed the level of IL-6 production of a series of OCCC cell lines. Among 5 cell lines, we chose RMG-1 cell with intermediate level of IL-6 production for further experiments (Supplementary Fig. S1A). Then, we examined the capacity of HUVECs to form tubular networks in the presence of conditioned medium from the OCCC cell line RMG-1 (Fig. 1A). Briefly, RMG-1 cells were cultured in 1% oxygen with or without anti-IL-6 antibody and the supernatant (conditioned medium) was used for tube formation assays. The ability of HUVEC cells to form tubular networks in RMG-1 conditioned media without IL-6 signal inhibition was equivalent to that in recombinant VEGF-supplemented culture medium as indicated by tube area measurements (Fig. 1A,B). As expected, the addition of Bev strongly impeded tube formation both in VEGF-supplemented medium and RMG-1 conditioned medium without IL-6 signal inhibition, suggesting that RMG-1 cells may also secrete VEGF (Fig. 1B). However, the efficacy of Bev to impede tube formation was strongly mitigated by RMG-1 condition medium with anti-IL-6 antibody, from 66 ± 3.8% in the absence of IL-6 signal blockade to only 29% ± 5.0% in the presence of anti-IL-6 (*p* < 0.001) (Fig. 1C). Moreover, this mitigation by anti-IL-6 antibody was dose dependent (Supplementary Fig. S2A,B).

### Attenuation of Bev anti-angiogenic and anti-tumor efficacy under IL-6 signal blockade

**Figure 2:**
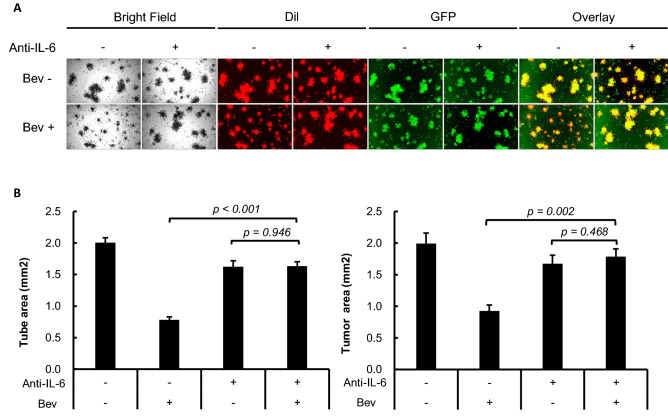
The role of IL-6 signal for the anti-angiogenic and the anti-tumor effects of Bev in 3D co-culture system. (**A**) Representative micrograph of 3D co-culture with or without IL-6 blockade and/or Bev observed through light microscope and fluorescent microscope at experimental day 4. HUVEC was stained by Dil, emitting red fluorescence. RMG-1/GFP was seen as green fluorescence. Overlaid images of Dil and GFP were shown at the rightmost. (**B**) Measured area at day 4 of each color by hybrid cell count software. Dil-stained area, regarded as the tube area, is shown on the left and GFP area, regarded as the tumor area, is shown on the right side. Error bars are SEs. All the images were observed by fluorescence microscope (BZ-X800, Keyence). Then, these obtained images were analyzed by the BZ-H4C analytic application (Keyence) for hybrid cell count and the BZ-H4CM application (Keyence) for macro cell count.

Findings from Fig. 1 suggest that IL-6 signaling potentiates the anti-angiogenic efficacy of Bev. To clarify the underlying mechanisms as well as the potential effects of IL-6 signaling on Bev anti-tumor activity, we examined these drugs in a co-culture system of the OCCC cell line RMG-1/GFP (emitting green fluorescence) and HUVEC pre-stained with Dil (emitting red fluorescence) (Fig. 2A). In this system, the tumor cell line attached to HUVEC and demonstrated proliferation, implying a direct interaction between the two (Supplementary Fig. S3A). Consistent with the results shown in Fig. 1, Bev treatment alone for 4 days reduced the tube area and this anti-angiogenic effect was significantly mitigated by anti-IL-6 (*p* < 0.001). Thus, reversal of Bev-mediated suppression of angiogenesis by IL-6 signal blockade was maintained in the presence of tumor cells. Moreover, the tumor area was also significantly larger in the combined Bev and anti-IL-6-treated group than in the groups treated with Bev alone (*p* = 0.002) (Fig. 2B), suggesting that IL-6 blockade disrupted both the anti-angiogenic and concomitant anti-tumor effects of Bev. Both effects of IL-6 signal blockade on Bev activity were observed over the entire 7-days experimental period (Supplementary Fig. S3B,C).

### IL-6 signaling blockade enhanced Ang1 release from tumor cells

**Figure 3:**
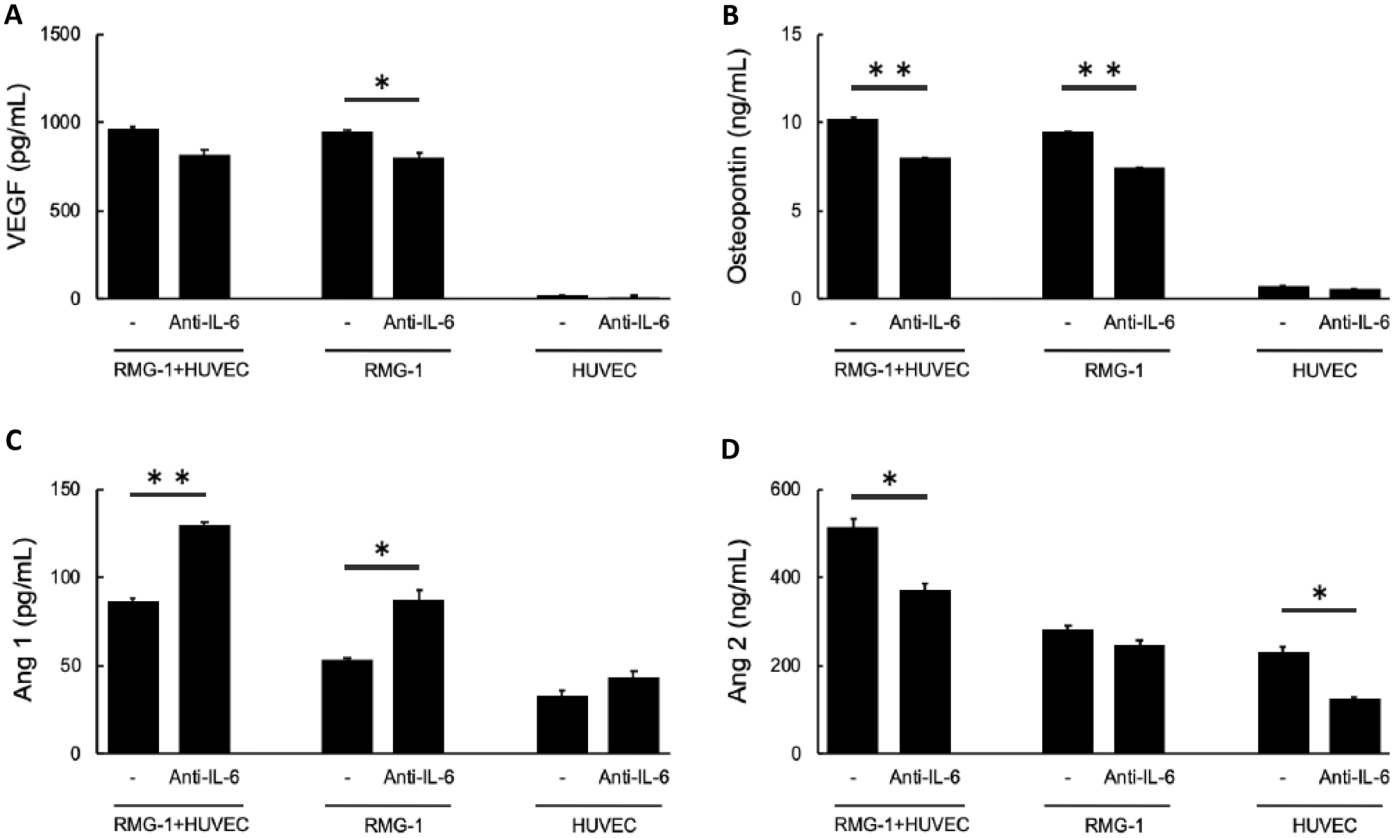
Angiogenic factors released by RMG-1 and HUVEC. (**A**) VEGF, (**B**) osteopontin, (**C**) Ang1, and (**D**) Ang2 production from RMG-1 and HUVEC in response to IL-6 signal blockade determined by ELISA assay. Data of HUVEC was calculated by the subtraction the data of RMG-1 mono-culture from that of RMG-1 + HUVEC. Error bars are SEs. *P < 0.05, **P < 0.005.

To further investigate the mechanisms underlying IL-6-dependent suppression of Bev anti-angiogenic activity, we examined the effects of IL-6 on secretion of the angiogenic modulators VEGF, Ang1/2, and osteopontin by tumor and HUVEC. Production of VEGF by RMG-1 cells was only mildly suppressed by anti-IL-6 antibody treatment as evidenced by ELISA analysis of culture supernatant (Fig. 3A), whereas osteopontin production by RMG-1 tumor cells was significantly suppressed by anti-IL-6 (*p* < 0.01) (Fig. 3B). In contrast, anti-IL-6 antibody treatment enhanced Ang1 production (*p* < 0.05) (Fig. 3C). HUVECs did not produce detectable VEGF or osteopontin, while Ang1 production was increased and Ang-2 production decreased by anti-IL-6 antibody treatment (Fig. 3A–D). These results suggest that the anti-angiogenic activity of Bev is mitigated by IL-6 signal blockade through enhanced production of Ang1 (rather than VEGF production). To further assess IL-6 effects on Ang1 production by OCCC cells, these ELISA assays were repeated on several other OCCC cell lines. In general, these assays indicated that IL-6 and Ang1 production are reciprocally regulated. While OVTOKO and HAC-2 lines produced little IL-6, both produced high levels of Ang1. Conversely, the OVISE line produced a large amount of IL-6 and a small amount of Ang1 (Supplementary Fig. S1A). Only RMG-1 and -2 cells produced moderate amounts of both IL-6 and Ang1. Moreover, anti-IL-6 antibody treatment enhanced Ang1 production in RMG-1, RMG-2, and OVISE cells (Supplementary Fig. S1B).

We then examined the relationship between IL-6 and Ang1 expression in 60 OCCC clinical tissue samples. Of the 13 tumor samples with high IL-6 expression, only one sample exhibited high Ang1 expression as evidenced by immunohistochemical staining. Further, Ang1 expression was significantly lower among tumor samples with high IL-6 expression than in samples with low IL-6 expression (*p* = 0.021) (Supplementary Fig. S4A,B). This result suggested that Bev may be more efficacious in tumors with high IL-6 expression because of the suppressive effect of IL-6 on pro-angiogenic Ang1 release.

### Suppression of Ang1 release by targeted siRNA restored the anti-angiogenic effects of Bev

**Figure 4:**
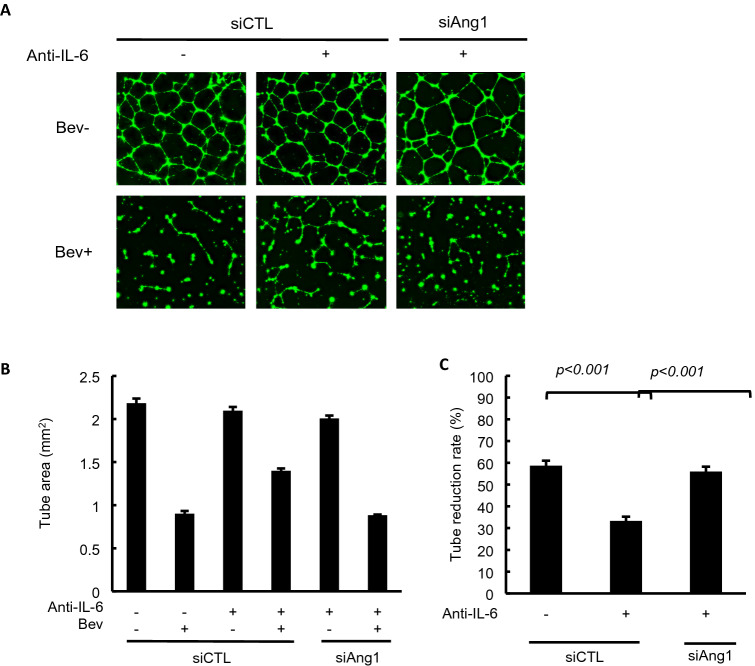
The role of Ang1 in IL-6 mediated Bev anti-angiogenic effect enhancement. (**A**) Images of tubes formed by HUVEC in the conditioned media from RMG-1 treated by indicated siRNA and anti-IL-6 antibody (magnification × 40). Fluorescent microscope observation was made after 18 h of incubation and Calcein AM staining. (**B**) Tubes in each well with indicated condition were measured by hybrid cell count software in order to elucidate the tube area. Data were average of triplicated well. (C) Reduction rate of tube area by Bev treatment with or without IL-6 blockade and siRNA, IL-6 blockade weakened Bev function in siCTL whereas siAng1 restored the anti-angiogenic function. Data are shown from one of two independent experiments with similar results. siCTL: siRNA without gene silencing ability (control). siAng1: angiopoietin-1 silencing siRNA. Error bars are SEs. All the images were observed by fluorescence microscope (BZ-X800, Keyence, Osaka, JAPAN). Then, these obtained images were analyzed by the BZ-H4C analytic application (Keyence) for hybrid cell count and the BZ-H4CM application (Keyence) for macro cell count.

To confirm that IL-6 potentiates the anti-angiogenic efficacy of Bev by suppressing Ang1 release, we conducted tube formation assays after manipulating Ang1 expression using targeted and control siRNAs. First, we confirmed the feasibility of this strategy by demonstrating that the targeted siRNA (siAng1) suppresses the production of Ang1 from RMG-1 with or without IL-6 signaling blockade (Supplementary Fig. S5). In the presence of conditioned medium from RMG-1 cells transfected with siAng1 and treated with anti-IL-6 antibody, Bev reduced the tube area by 55%, similar to the findings in assays using conditioned medium from tumor cells that were not treated with anti-IL-6 (Fig. 4A–C). In other words, suppression of Ang1 accumulation in the conditioned medium restored the anti-angiogenic efficacy of Bev in the presence of anti-IL-6, providing further evidence that IL-6 indirectly promotes the anti-angiogenic activity of Bev by suppressing Ang1 secretion by OCCC cells.

## Discussion

Molecular profiling of OCCC tumor tissues has revealed several potential prognostic biomarkers, predictors of treatment response, and therapeutic targets^9–14^. For instance, studies have reported the hyperactivation of several signaling pathways, including hypoxia-inducible factor 1α (HIF-1α)/VEGF and IL-6/STAT3 pathways in OCCC^15–17^. Mabuchi et al. reported that OCCC cells under intratumoral hypoxia strongly expressed VEGF and that Bev demonstrated anti-tumor efficacy against OCCC both in vitro and in vivo^18^. However, they also suggested that VEGF may not be a reliable biomarker for predicting Bev sensitivity. Therefore, although anti-angiogenic treatment by Bev appears promising based on pathogenesis, there is still no widely recognized biomarker predictive of its clinical efficacy. Here we demonstrate that high IL-6 or low Ang1 may be such predictors.

Upregulation of IL-6 and related pathway mediators as well as the anti-tumor efficacy of IL-6 pathway inhibition have been reported in OCCC^5,6,19,20^. In addition, several studies have documented significant associations between poor OCCC prognosis and high IL-6 in tumor or serum samples^5,11,20^. Anglesio et al. reported upregulation of the IL-6/STAT3/HIF pathway and therapeutic responses to the anti-angiogenic agent sunitinib in two chemotherapy-resistant OCCC cases^5^. However, the phase II GOG-254 trial evaluating this multi-receptor tyrosine kinase inhibitor for the treatment of persistent or recurrent OCCC found minimal clinical efficacy^21^. Recently, a retrospective biomarker analysis revealed longer survival by Bev-treated patients with high plasma IL-6 level compared to those with lower plasma IL-6^8^. Based on these findings, we evaluated the influence of IL-6 on anti-angiogenic and anti-tumor efficacies of Bev using an in vitro OCCC model to reveal potential molecular mechanisms, and further examined the association of IL-6 expression with that of the pro-angiogenic factor Ang1 among OCCC patients to help identify good candidates for Bev treatment.

**Figure 5:**
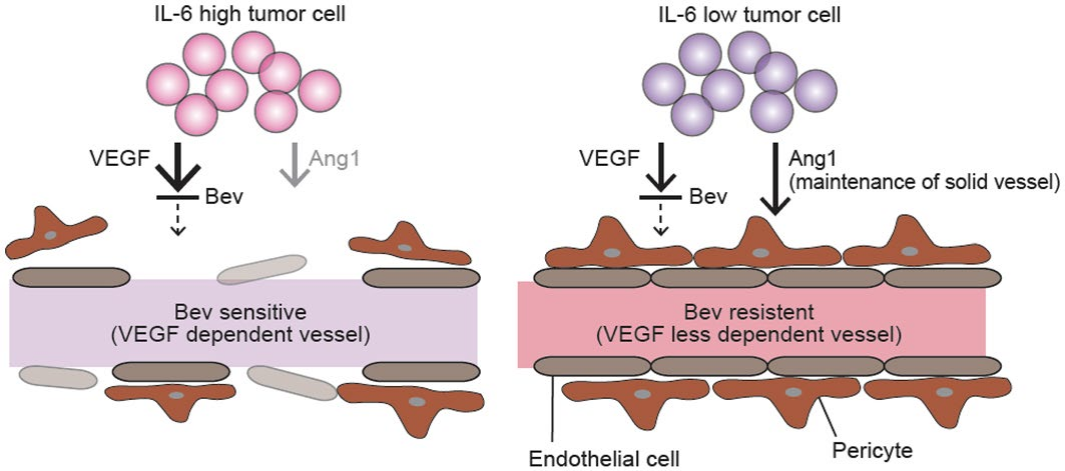
Mechanism of enhanced anti-angiogenic efficacy of Bev in IL-6 high tumor with Ang1 suppression. IL-6 supports the anti-angiogenic activity of Bev by suppressing Ang1 expression and promoting dependence on VEGF for angiogenesis. (This illustration is drawn by means of Adobe Illustrator).

In general, IL-6 has been demonstrated to influence angiogenesis as a pro-angiogenic factor^22^. For instance, transgenic mice engineered to overexpress IL-6 exhibited hypervascularization of the cerebellum^23^, whereas IL-6–deficient mice exhibited reduced angiogenic responses to wound injury^24^. Further, IL-6 induced VEGF production by tumor cells and consequently activated angiogenesis^25,26^. In the current study, IL-6 signaling blockade reduced the anti-angiogenic activity and associated anti-tumor activity of Bev in vitro co-culture system of OCCC cells and HUVECs. However, the moderate alteration of VEGF production by OCCC cells under IL-6 blockade suggested that some other angiogenic factor(s) were responsible for the observed attenuation of the anti-angiogenic activity of Bev. Several VEGF-independent angiogenic mechanisms of IL-6 have been reported, including Ang1 modulation, and Kayakabe et al. reported that IL-6 could destabilize angiogenesis by inhibiting Ang1 signaling in a co-culture model of rheumatoid arthritis^27^. Additional analyses in our model suggested that IL-6 promoted the anti-angiogenic activity of Bev by suppressing Ang1 release from OCCC cells. Furthermore, the association of high IL-6 expression with low Ang1 expression was found in a series of specimens from patients with OCCC. According to these results, Ang1 may interfere with the function of Bev in OCCC (Fig. 5).

Ang1 is believed to facilitate vessel stabilization, and its signaling is believed to support the induction and growth of tumor vasculature even under VEGF blockade, resulting in improved tumor perfusion^28^. Huang et al. also reported that Ang1 protected the tumor vasculature from regression, increased vessel caliber, and induced the recruitment of mural cells under anti-VEGF treatment^29^. Moreover, Casanovas et al. found that tumors exhibiting continued progression during anti-VEGF receptor-2 antibody treatment maintained substantial Ang1 expression^30^. According to these findings, Ang1 suppression may be important for Bev to exert its effects. In the current study, we found that IL-6 blockade enhanced Ang1 production by tumor cells, whereas siRNA-induced Ang1 depletion restored the impaired anti-angiogenic function of Bev under IL-6 blockade. Collectively, IL-6 signaling could reduce the pro-angiogenic function of Ang1 in tumors and relatively enhance that of VEGF, which in turn enhances Bev function in OCCC cells.

The utility of IL-6 as a potential predictor of anti-angiogenic drug response is controversial. Earlier reports suggested that high IL-6 levels could be predictive of a survival benefit of Bev treatment in patients with EOC^8^, metastatic renal cancer^31^, and metastatic colorectal cancer^10^. Conversely, other studies reported that low IL-6 levels were associated with better Bev treatment responses in hepatocellular carcinoma^32^, pancreatic cancer^33^, and metastatic colorectal cancer^34^. In addition, a recent clinical study found that aflibercept, a recombinant fusion protein that blocks the VEGF pathway in advanced EOC, was more effective in patients with low IL-6 levels^35^. Differences in cellular IL-6 responses among tumor types, study design, or clinical stage may explain these discordant results. Further research is warranted to elucidate the mechanism by which IL-6 modulates Ang1 and the prognostic utility of IL-6 as a biomarker of anti-angiogenic drug response.

In conclusion, this study demonstrated that IL-6 enhanced the anti-angiogenic efficacy of Bev by suppressing Ang1 in addition to increasing VEGF production in OCCC cells. Furthermore, the current study provides a strong rationale for prospective clinical trials of anti-angiogenic therapy for EOC, including OCCC, to clarify the prognostic utility of IL-6 and Ang1 levels.

## Materials and methods

### Cell culture

The human OCCC cell lines RMG-1 and RMG-2 were kindly provided by Dr. D. Aoki (Keio University, Tokyo, Japan) and maintained in Ham’s F12 (GIBCO BRL, Grand Island, NY, USA) supplemented with 10% fetal bovine serum (FBS) (Cytiva, Marlborough, MA, USA). The OVTOKO and OVISE lines were purchased from the Japanese Collection of Research Bioresources Cell Bank (Osaka, Japan), and the HAC-2 line was kindly provided by Dr. M. Nishida (Tsukuba University, Tsukuba, Japan). These three cell lines were maintained in RPMI-1640 medium (Sigma–Aldrich, St Louis, MO, USA) supplemented with 10% FBS. For experiments, all OCCC cell lines were pre-incubated for 24 h in M199 medium (Sigma–Aldrich) supplemented with 0.5% bovine serum albumin (BSA) (Sigma–Aldrich) under a hypoxic environment (1% O_2_) created by the BIONIX hypoxic culture kit (SUGIYAMA-GEN, Tokyo, Japan). To modulate IL-6 signaling, the indicated concentration of anti-IL-6 antibody (MAB206-100, clone 6708; R&D Systems, Minneapolis, MN) was added to the medium and culture continued for 48 h. Other cultures were incubated for the same period but without anti-IL-6 treatment. Subsequently, supernatants were collected as conditioned media (with or without anti-IL-6) and stored frozen for further experiments. An RMG-1 line expressing green fluorescent protein (RMG-1/GFP) was purchased from Anticancer Japan (Chiba, Japan) and treated in the same manner as RMG-1 cells. HUVEC cells were kindly provided by Dr. I. Yamamoto (The Jikei University School of Medicine, Tokyo, Japan) and maintained in endothelial cell medium (Sciencell Research Laboratory, Carlsbad, CA) with 5% FBS.

### Transfection and siRNA

A predesigned small interfering RNA (siRNA) targeting angiopoietin-1 (siAng1) (Silencer Select Pre-Designed siRNA, Assay ID: s1356) and a non-targeted control siRNA (siCTL) (Silencer Select Negative Control, #4390843) were purchased from ThermoFisher Scientific (Waltham, MA). Prior to transfection, siRNAs were premixed with lipofectamine RNAiMAX (ThermoFisher Scientific) plus Opti-MEM (ThermoFisher Scientific). RMG-1 cells were then transfected by incubation with this mixture for 24 h under a hypoxic environment. After an additional 24 h of incubation, the culture medium was exchanged for fresh medium with or without 100 ng/mL anti-IL-6 antibody as indicated. The supernatants from these cultures were collected as conditioned media for tube formation assays.

### Antibodies and angiogenic factor

Recombinant human VEGF (aa207-318) was purchased from PeproTech (Rocky Hill, NJ). The human VEGF-A monoclonal antibody Bev was kindly provided by Chugai Pharmaceutical Co., Ltd (Tokyo, Japan).

### Tube formation assay

Tube formation assays were conducted as described by Yin et al.^36^. Briefly, Corning 96-well flat bottom plates (Corning, NY) were coated with 30 μL growth factor-reduced Matrigel matrix (Corning) and seeded with 1 × 10^5^ HUVEC in 100 μL RMG-1 conditioned medium supplemented with 0.5% BSA. Recombinant VEGF (5 ng/mL) and Bev (10 µg/mL) were added as indicated for specific experiments. After 18 h of incubation, tubes were stained with Calcein AM (Corning) to quantify total tube area using a fluorescence microscope (BZ-X800, Keyence, Osaka, JAPAN) and the BZ-H4C analytic application (https://www.keyence.com/products/microscope/fluorescence-microscope/bz-x700/models/bz-h4c/) for hybrid cell count and the BZ-H4CM application (https://www.keyence.com/products/microscope/fluorescence-microscope/bz-x700/models/bz-h4cm/) for macro cell count.

### Enzyme-linked immunosorbent assays (ELISAs)

ELISAs were performed to measure the medium concentrations of angiogenic factors secreted by RMG-1, RMG-2, OVISE, OVTOKO, and HAC-2 cell lines. Briefly, cells were seeded in T-25 flasks at 5 × 10^5^ cells per flask. After 24 h of incubation, the culture medium was exchanged for M199 medium plus 0.5% BSA with or without anti-IL-6 antibody (100 ng/mL) as indicated and incubation was continued under hypoxia for an additional 48 h. Part of the culture supernatant was then collected for ELISA analysis of VEGF, IL-6, Ang1, angiopoietin-2 (Ang2), and osteopontin concentrations using specific ELISA kits (DVE00 for VEGF, D6050 for IL-6, DANG10 for Ang1, DANG20 for Ang2, and DOST00 for osteopontin; all from R&D Systems). The remaining supernatants were collected as conditioned media for HUVEC culture. HUVEC were initially seeded at 1 × 10^5^ cells per T-25 flask. After 24 h of incubation, the culture medium was exchanged for RMG-1 cell-conditioned medium and incubation continued for an additional 48 h. The supernatants were then collected for analysis using the same ELISA kits.

### Co-culture assay

A 3D-culture protocol was performed based on the Matrigel sandwich structure method described previously^37^. Briefly, 30 μL Matrigel was added into each well of a 96-well plate pre-chilled on ice. The plates were then incubated at 37 °C for 30 min to enable polymerization of this Matrigel basal layer. HUVEC cells were pre-stained with Corning DilC12(3) Fluorescent Dye (Dil), suspended at 2 × 10^4^ cells per 75 µL M199 medium supplemented with 1% FBS, and seeded onto the basal layer. After 4 h of incubation to allow tube formation, the same number of RMG-1/GFP cells suspended in 75 µl pre-chilled M199 medium containing 1% FBS, 10 µg/mL Bev, and 10% Matrigel with or without 100 ng/mL anti-IL-6 antibody were seeded onto the polymerized Matrigel base layer. Then, the plates were incubated at 37 °C to allow polymerization of the top layer. The co-culture system was maintained in M199 medium containing 1% FBS at 37 °C under a 5% CO_2_ atmosphere for 7 days with medium exchange every other day. The Dil-stained area and GFP-stained area, corresponding to tube area and tumor area, respectively, were quantified using a fluorescence microscope (BZ-X800, Keyence) and the BZ-H4C and BZ-H4CM analytic applications.

### Clinical samples and immunohistochemistry

Analysis of human tumor samples was approved by the ethics committee of The Jikei University School of Medicine (32-017(10092)). Sixty stage II‒IV OCCC patients receiving primary surgical resection followed by post-operative adjuvant chemotherapy at The Jikei University School of Medicine and affiliated hospitals from 2013 to 2018 were enrolled. Paraffin-embedded tumor samples from primary surgery were stained with hematoxylin–eosin to confirm the diagnosis of OCCC. To examine IL-6 and Ang1 expression, formalin-fixed, paraffin-embedded tissue sections (4 μm thick) were deparaffinized, incubated in Cell Conditioning 1 (CC1) standard solution (citrate buffer pH 8.5, Ventana Medical Systems) for 60 min at 100 °C for antigen retrieval, and then incubated in antibodies against IL-6 (1:400, 21865-1-AP; Proteintech) and Ang1 (1:200, ab8451; Abcam, Cambridge, UK). Immunostained slides were evaluated by two independent pathologists (H.M and M.M) blinded to clinical information. Staining scores were standardized by comparison between the observers, and discrepancies were resolved by reevaluating the slides using a multi-head microscope. Immunoreactivity for IL-6 in the tumor cytoplasm was categorized as none/focal (0%–40%) or diffuse (50%–100%)^11^. Immunostaining for Ang1 was assessed in a semi-quantitative manner as previously described^38^. Tumor and stromal areas were evaluated independently and each section was assigned two scores: Staining intensity (0, no staining; 1, weak staining; 2, moderate staining; 3, intense staining) and proportion of cells stained (0, no cells staining; 1, 1%–25%; 2, 26%–50%; 3, 51%–75%; 4, 76%–100%). The scores for staining intensity and proportion of stained cells were multiplied to yield individual tumor and stromal area staining scores for Ang1. The two scores were then added to yield a final Ang1 staining score for each sample, which was then categorized as high (> 8) or low (0–7).

### Statistical analysis

All data are expressed as the mean ± standard error of the mean. Means of experimental and control groups were compared by independent samples Student’s t-test, one-way analysis of variance followed by post hoc Bonferroni’s multiple comparison tests, or Fischer’s exact tests as indicated. A p < 0.05 (two-tailed) was considered statistically significant for all tests. All statistical analyses were performed using EZR software (Saitama Medical Centre, Jichi Medical University; http://www.jichi.ac.jp/saitama-sct/SaitamaHP.files/ statmedOSX.html, Kanda, 2012), a graphical interface for R (The R foundation for Statistical Computing. Vienna, Austria, ver. 3.2.2).

## Supplementary Information

Supplementary Information.
