# Supplementary material for: Interleukin-6 as an enhancer of anti-angiogenic therapy for ovarian clear cell carcinoma
Source: Sci Rep. 2021 Apr 8;11:7689. doi: 10.1038/s41598-021-86913-9 (PMC8032732; doi:10.1038/s41598-021-86913-9)
Supplement: Supplementary file 1 — Supplementary Information. [file 41598_2021_86913_MOESM1_ESM.pdf]

Supplementary figures

Interleukin-6 as an Enhancer of Anti-angiogenic Therapy for Ovarian Clear Cell Carcinoma

Toshiyuki Seki[1 §], Nozomu Yanaihara[1 § *], Jason Solomon Shapiro[1, 2], Misato Saito[1], Junya Tabata[1], Ryo Yokomizo[1], Daito Noguchi[1], Takafumi Kuroda[1], Ayako Kawabata[1], Jiro Suzuki[1], Kazuaki Takahashi[1], Haruka Matsuzawa[3], Misayo Miyake[3], Masataka Takenaka[1], Yasushi Iida[1], Satoshi Yanagida[1], and Aikou Okamoto[1]

[1]Department of Obstetrics and Gynecology, The Jikei University School of Medicine, Tokyo, Japan

[2]Feinberg Cardiovascular Research Institute, Northwestern University, Chicago, IL 60611, USA

[3]Department of Pathology, The Jikei University School of Medicine, Tokyo, Japan

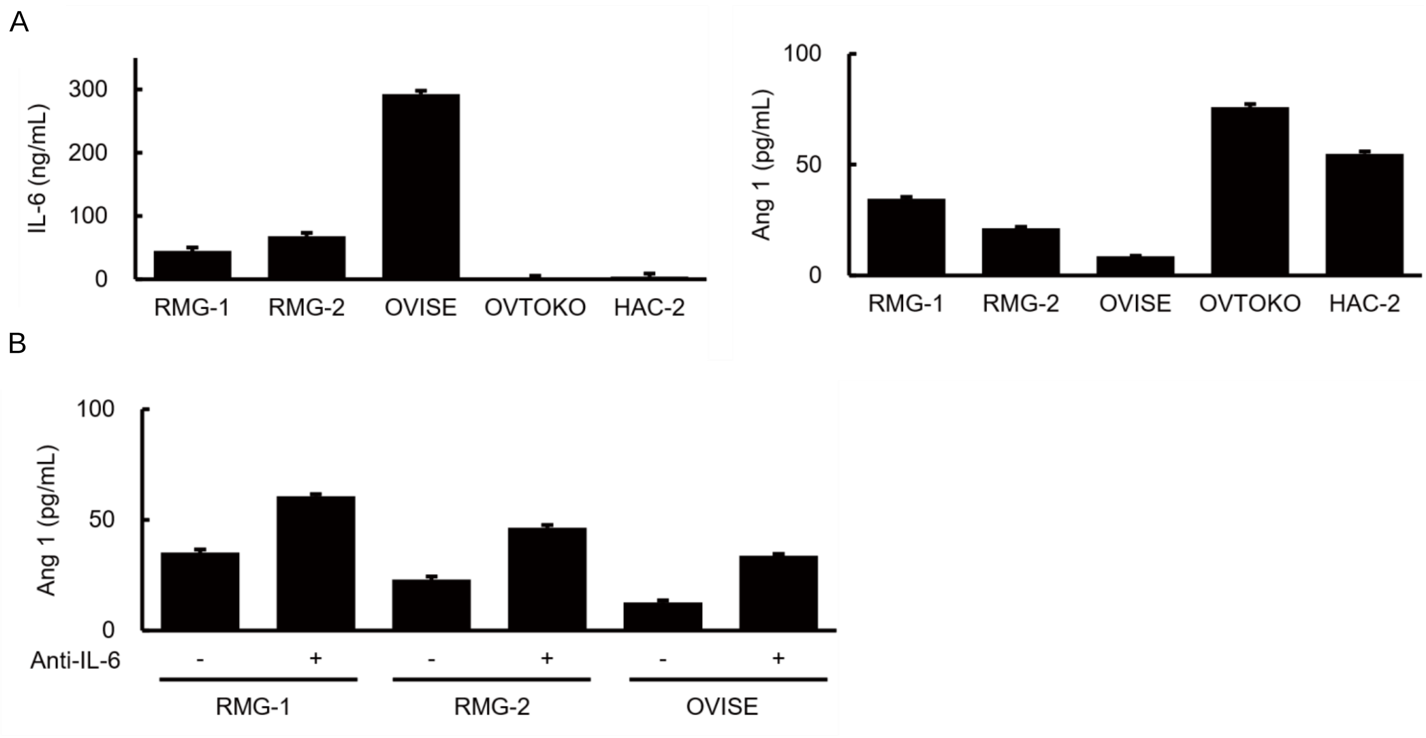

Supplementary Figure S1. The expression IL-6 and Ang1 in OCCC cell lines. (A) IL-6 (left) and Ang1(right) levels in the culture supernatant of OCCC cell lines (RMG-1, RMG-2, OVISE, OVTOKO, and HAC-2). (B) Ang1 levels in the culture supernatant of anti-IL-6 antibody treated OCCC cell lines (RMG-1, RMG-2, OVISE). Error bars are SEs**.**

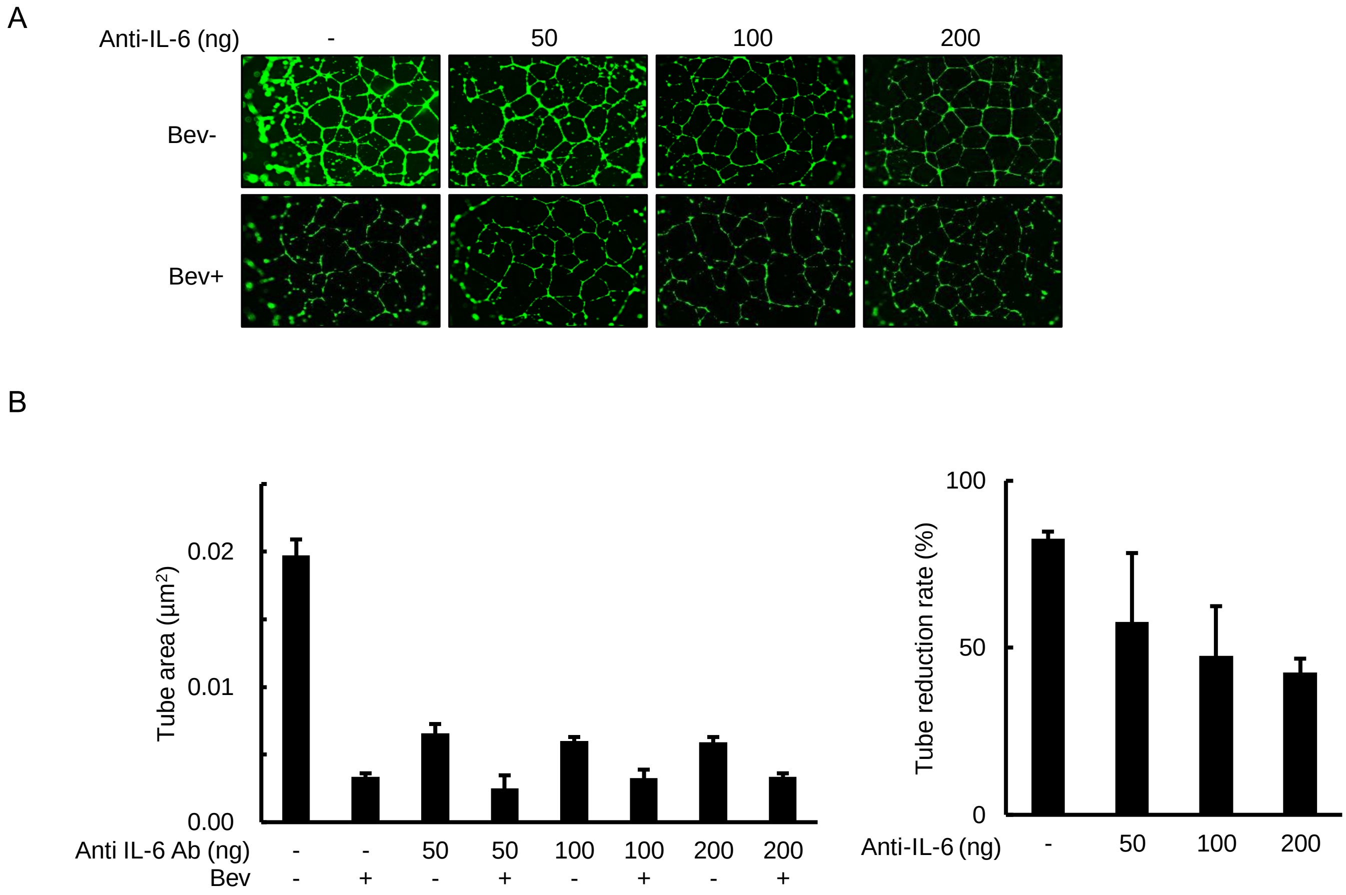

Supplementary Figure S2. Tube formation assay, HUVEC cultured under RMG-1 conditioned media with different concentration of anti-IL-6 antibody. (A) Representative images of tube formation of HUVEC with the indicated reagents (magnification ×100). Fluorescent microscope observation was made after 18 hours of incubation and Calcein AM staining. (B left) Tube area in each well with indicated concentrations of anti-IL-6 antibody were measured by hybrid cell count software. Data are represented as average of triplicated well. (B right) Reduction rate of tube area by bevacizumab treatment with indicated concentrations of anti-IL-6 antibody. Data are shown from one of two independent experiments with similar results. Error bars are SEs. All the images were observed by fluorescence microscope (BZ-X800, Keyence) and analyzed by the BZ-H4C analytic application (Keyence) for hybrid cell count and the BZ-H4CM application (Keyence) for macro cell count.

A

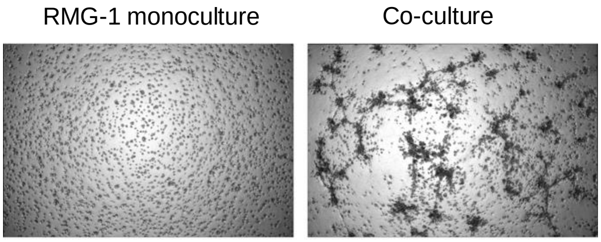

B

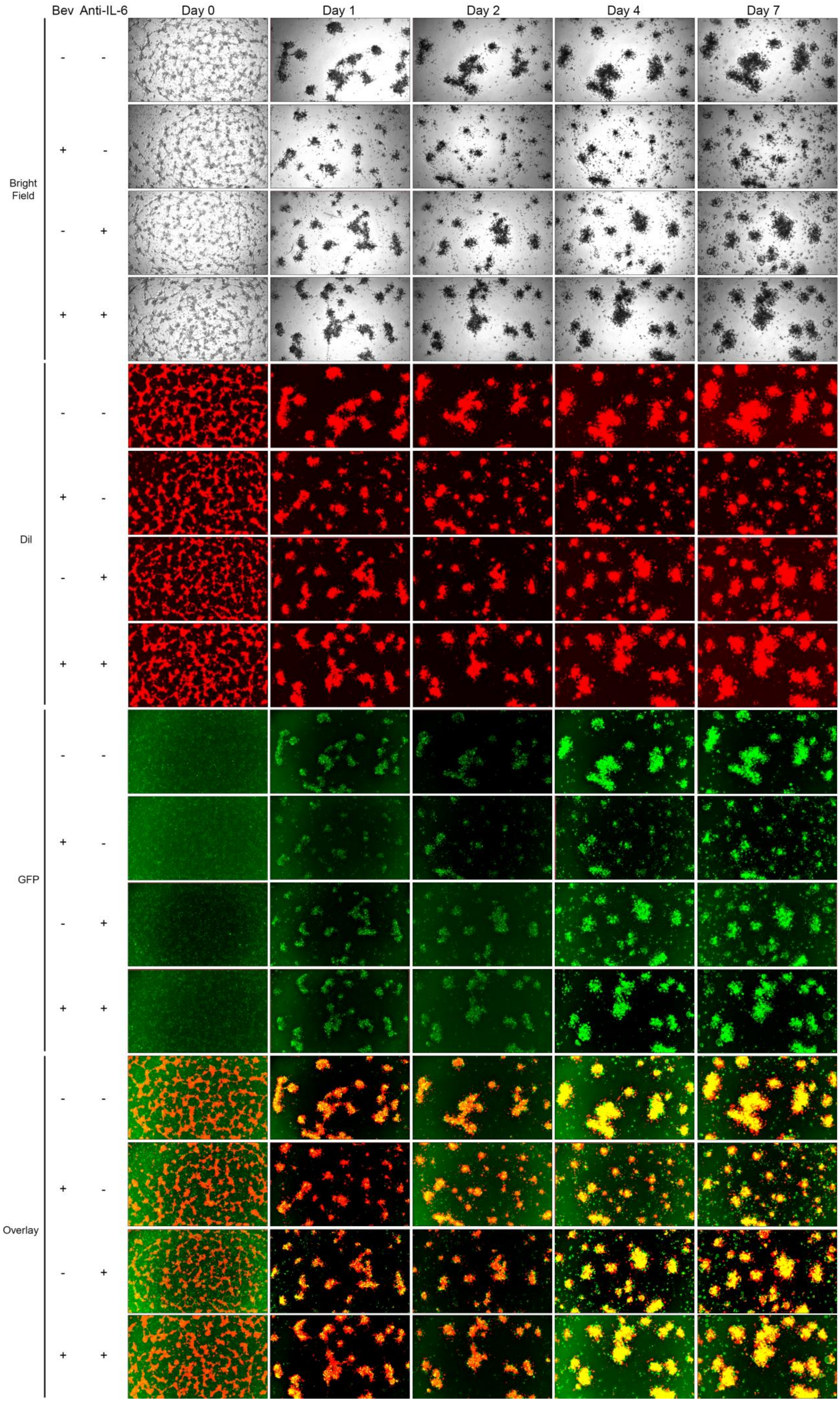

Bev
Anti-IL-6
Day 0
Day 1
Day 2
Day 4
Day 7
Bright Field
DiI
GFP
Overlay
- -
+ -
- +
+ +

C

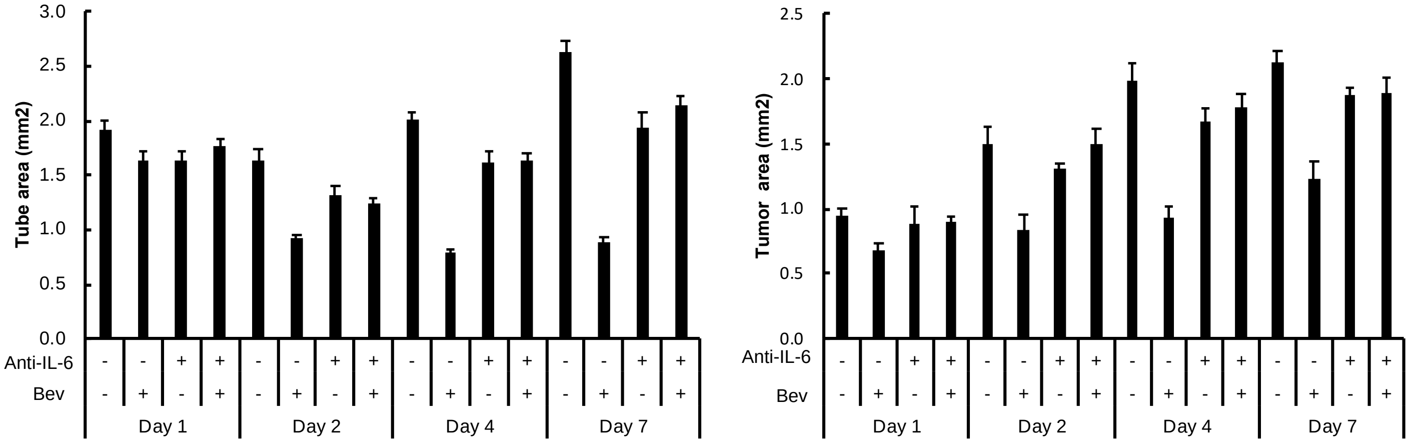

Supplementary Figure S3. 3D coculture assay. (A) Representative micrograph of 3D co-culture of RMG-1 and HUVEC at experimental day 4 (no treatment). (B, C) Time series of tube formation and tumor growth in co-culture system. (B) Representative images of co-culture in the indicated reagents (magnification ×40). Fluorescent microscope observation was made at experimental day 0, 1, 2, 4, and 7. Dil stained HUVEC cell showed red color and RMG-1/GFP cell showed green color. (C left) Tube area in each well with or without anti-IL-6 antibody and Bev at each time point. Data were average of triplicated well. (C right) Tumor area in each well with or without anti-IL-6 antibody and Bev at each time point. Data are represented as average of triplicated well. Data are shown from one of two independent experiments with similar results. Error bars are SEs. All the images were observed by fluorescence microscope (BZ-X800, Keyence) and analyzed by the BZ-H4C analytic application (Keyence) for hybrid cell count and the BZ-H4CM application (Keyence) for macro cell count.

A

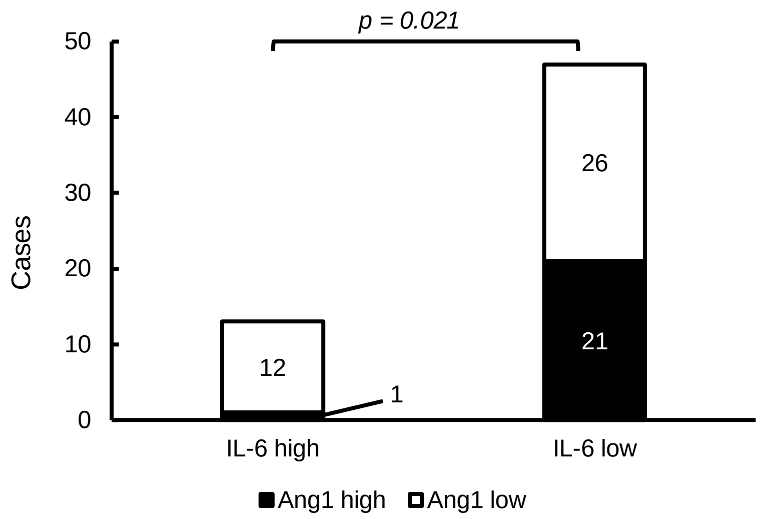

B

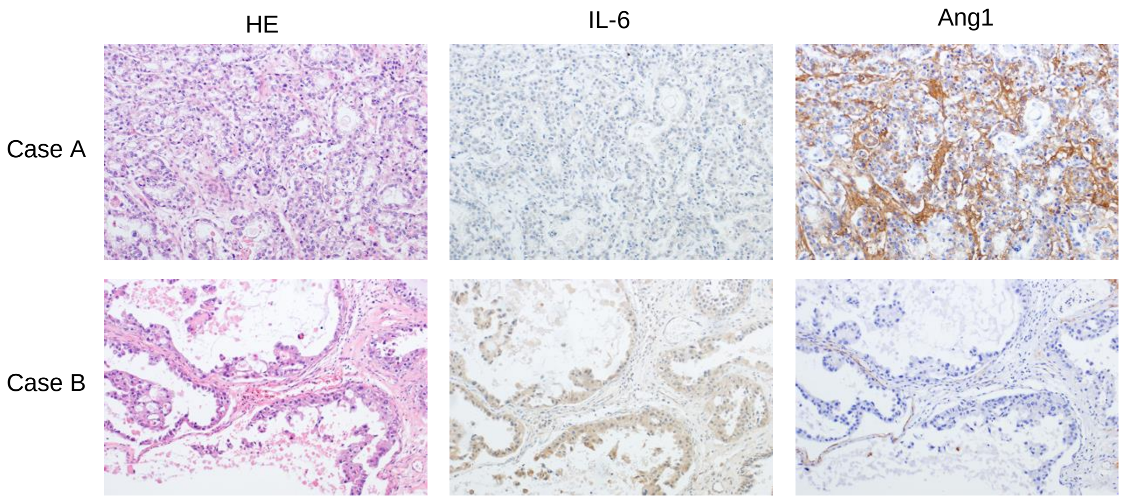

Supplementary Figure S4. The expression of IL-6 and Ang1 in clinical samples. Result of immunohistochemistry staining of OCCC patients' samples. (A) Frequency of Ang1 high and low patients in IL-6 high and low patients. (B) Representative slide images from 2 patients. One patient (case A) with IL-6 low and Ang1 high and the other (case B) with IL-6 high and Ang1 low.

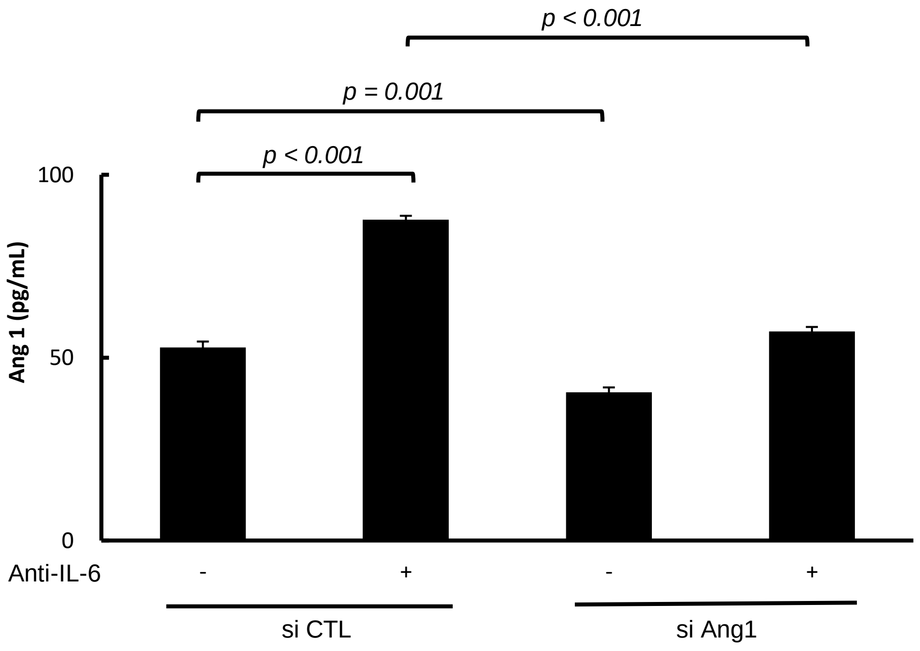

Supplementary Figure S5. Confirmation of gene silencing effect of siRNA. Ang1 concentration in culture supernatant of RMG-1 transfected with siCTL or siAng1 were measured by ELISA. Error bars are SEs.
